# Supplementary material for: Multi-year El Niño events tied to the North Pacific Oscillation
Source: Nat Commun. 2022 Jul 5;13:3871. doi: 10.1038/s41467-022-31516-9 (PMC9256710; doi:10.1038/s41467-022-31516-9)
Supplement: Supplementary file 1 — Supplementary Information [file 41467_2022_31516_MOESM1_ESM.pdf]

Supplementary Materials for

Multi-year El Niño events tied to the North Pacific Oscillation

This PDF file includes:

Supplementary Table 1. Observed multi-year El Niño/La Niña events for the period 1950–2020.

Supplementary Table 2. Observed single-year El Niño events for the period 1950–2020.

Supplementary Table 3. Description of the selected 29 models from the CMIP5/6 archives.

Supplementary Figure 1. Time series of the Niño3.4 index (°C) for the period 1950–2020.

Supplementary Figure 2. Forecasts of the Niño3.4 index respectively from February 2014 and February 2015 by operational forecasting models compiled at the IRI/CPC (International Research Institute for Climate and Society/Climate Prediction Center).

Supplementary Figure 3. The JFM(0) SLP anomalies prior to multi-year El Niño events in observations.

Supplementary Figure 4. Evolutions of SST and 850 hPa winds anomalies composited for multi-year El Niño events in observations.

Supplementary Figure 5. Evolutions of SST anomalies composited for multi-year El Niño events in observations and models.

Supplementary Figure 6. The atmospheric teleconnections of the CP and EP ENSO.

Supplementary Figure 7. Relationships among the NPO, SLP_{HI}, and NPMM indices.

Supplementary Figure 8. Evolutions of SST and SLP anomalies composited for multi-year El Niño events from JFM(0) to JFM(2) derived from different SST and SLP datasets.

Supplementary Figure 9. The JFM(1) anomalies of SST, SLP and 850 hPa winds for individual multi-year El Niño events.

Supplementary Figure 10. Evolutions of SST and SLP anomalies composited for the NPO-preceded single-year El Niño events in observations.

Supplementary Figure 11. Composite structures of JFM(1) SST anomalies for the

NPO-preceded and non-NPO-preceded CP El Niño events.

Supplementary Figure 12. Evolutions of SST and SLP anomalies composited for the observed CP El Niño events without the preceding NPO in observations.

Supplementary Figure 13. Effects of the NPO on the frequency ratio of multi-year El Niño/La Niña events in the CMIP5/6 models.

Supplementary Figure 14. The frequency ratio of the NPO-preceded El Niño/single-year El Niño events in the CMIP5/6 models.

Supplementary Figure 15. Evolutions of SST and SLP anomalies composited for the NPO-preceded multi-year El Niño events in the CMIP5/6 models.

Supplementary Figure 16. The imposed NPO forcing in numerical experiments.

Supplementary Figure 17. The JFM(0) SST anomalies prior to multi-year La Niña events in observations.

Supplementary Figure 18. Evolutions of multi-year La Niña events preceded by the negative NPO event alone in the CMIP5/6 models.

Supplementary Table 1. Observed multi-year El Niño/La Niña events for the period 1950–2020.

Multi-year El Niño events (5)	Multi-year La Niña event (7)
1957/58/59, 1968/69/70, 1986/87/88, 2014/15/16, 2018/19/20	1954/55/56, 1970/71/72, 1973/74/75, 1983/84/85, 1998/99/2000, 2007/08/09, 2010/11/12

Supplementary Table 2. Observed single-year El Niño/La Niña events for the period 1950–2020.

Single-year El Niño events (14)	Single-year La Niña event (6)
1951/52, 1963/64, 1965/66, 1972/73, 1976/77, 1977/78, 1982/83, 1991/92, 1994/95, 1997/98, 2002/03, 2004/05, 2006/07, 2009/10	1950/51, 1964/65, 1988/89, 1995/96, 2017/18, 2020/21

Supplementary Table 3. Description of the selected 29 models from the CMIP5/6 archives. The 23 CMIP5/6 models whose RCP8.5 simulations are available are indicated by the asterisk.

Archive	Model Number	Model name	Model center
**CMIP5**	1	ACCESS1-3*	CSIRO-BOM/Australia
	2	CMCC-CM*	CMCC/Italy
	3	CESM1-CAM5*	NSF-DOE-NCAR/United States
	4	CMCC-CMS*	CMCC/Italy
	5	CNRM-CM5*	CNRM-CERFACS/France
	6	FGOALS-g2*	LASG-IAP/China
	7	GFDL-CM3*	NOAA-GFDL/USA
	8	GISS-E2-H*	NASA-GISS/USA
	9	GISS-E2-R-CC*	NASA-GISS/USA
	10	GISS-E2-R*	NASA-GISS/USA
	11	IPSL-CM5B-LR*	IPSL/France
	12	IPSL-CM5A-MR*	IPSL/France
	13	MPI-ESM-MR*	MPI-M/Germany
**CMIP6**	14	ACCESS-ESM1_5*	CSIRO-ARCCSS/Australia
	15	AWI_CM_1_1_LR	AWI/Germany
	16	BCC_ESM1	BCC/China
	17	CAMS_CSM1_0*	CAMS/China
	18	CAS_ESM2_0*	CAS/China
	19	CESM2-FV2	NCAR/United States
	20	CIESM*	THU/China
	21	E3SM-1-1	E3SM-Project/United States
	22	GISS_E2_1_G*	NASA-GISS/USA
	23	GISS_E2_1_G_CC	NASA-GISS/USA
	24	HadGEM3-GC31-LL*	MOHC/UK
	25	IITM-ESM*	CCCR-IITM/India
	26	MCM-UA-1-0*	UA/USA
	27	MIRPC6*	MIROC/Japan
	28	MRI-ESM2-0*	SNU/Korea
	29	SAM0-UNICON	SNU/Korea

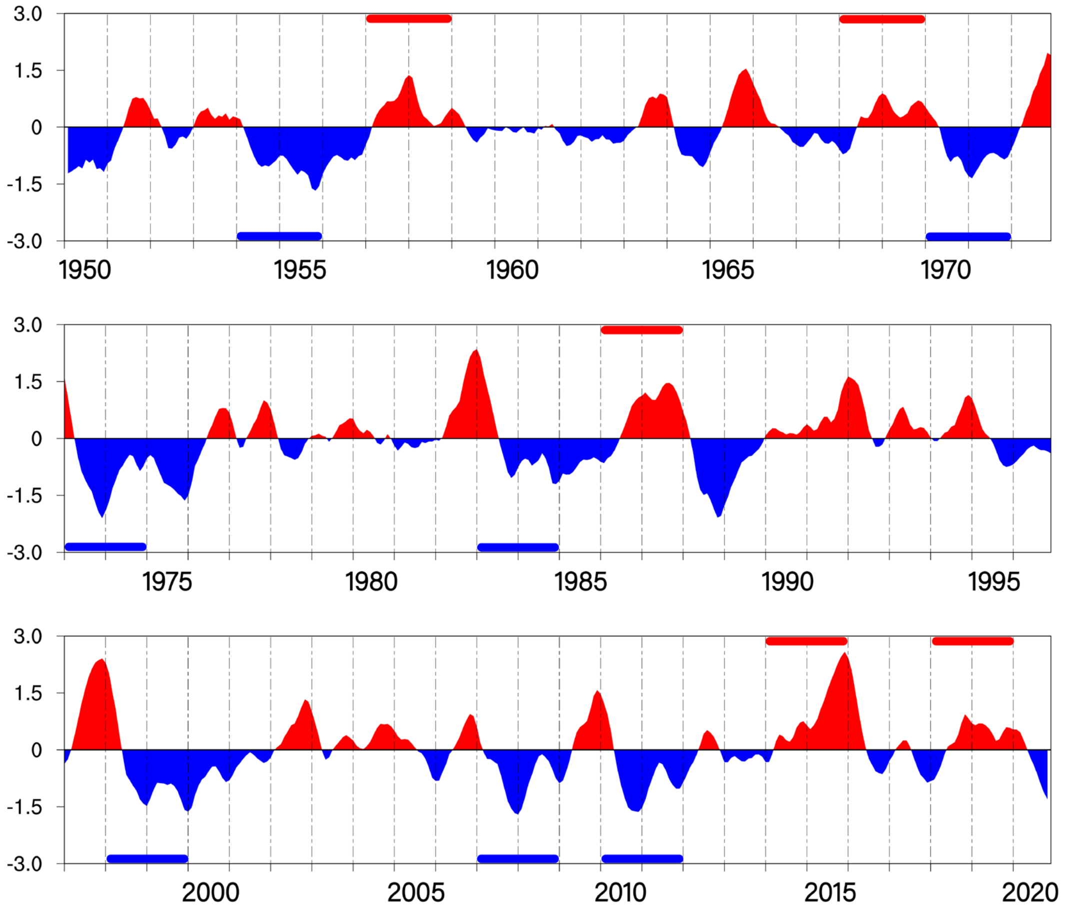

Supplementary Figure 1. Time series of the Niño3.4 index (°C) for the period 1950–2020. Years 0 and 1 of multi-year El Niño events (see Methods section for the definition of multi-year El Niño events) are indicated by thick horizontal red lines along the time axis, and years 0 and 1 of multi-year La Niña events are given by thick horizontal blue lines. The time series is smoothed with a 3-month running-mean filter.

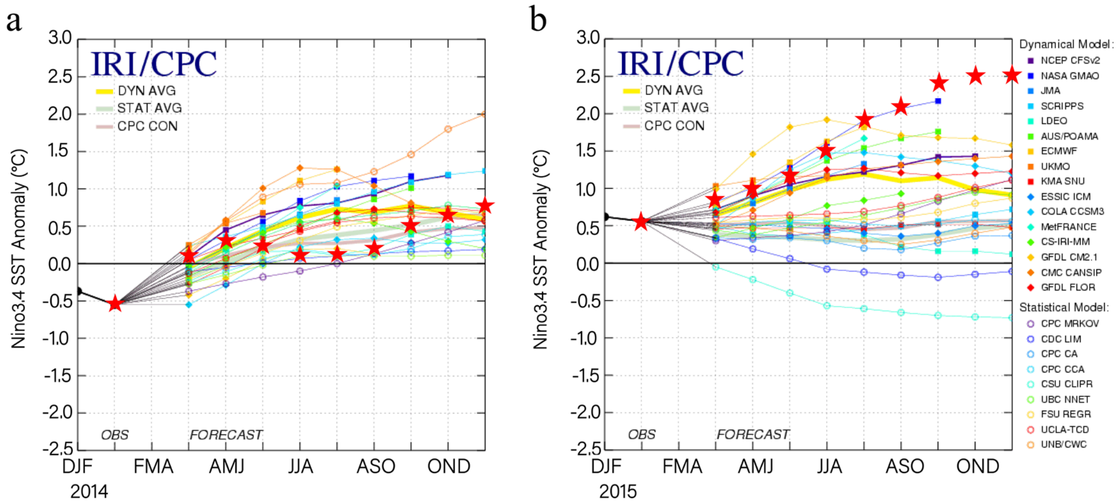

Supplementary Figure 2. Forecasts of the Niño3.4 index respectively from February 2014 and February 2015 by operational forecasting models compiled at the IRI/CPC (International Research Institute for Climate and Society/Climate Prediction Center). (**a**) Forecasts starting from February 2014. (**b**) Forecasts starting from February 2015. In (**a,b**), observations are indicated by the large red star.

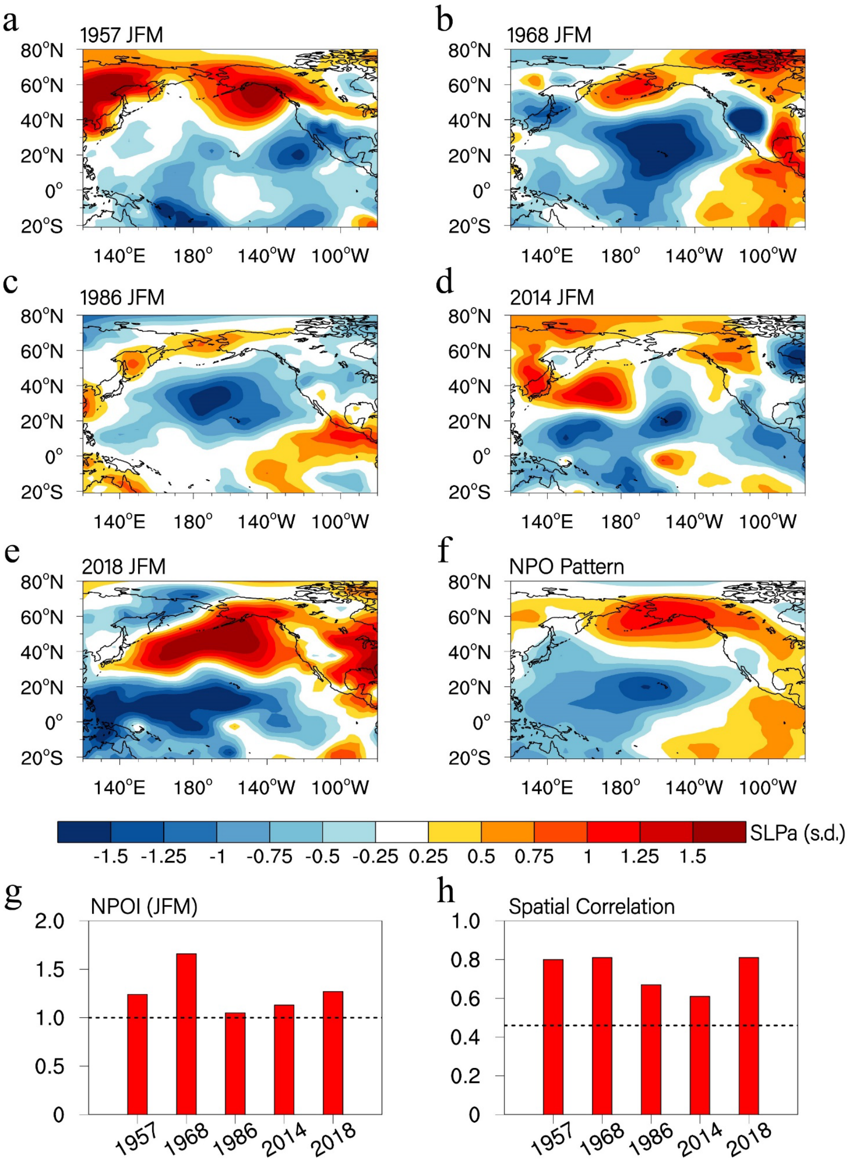

Supplementary Figure 3. The JFM(0) SLP anomalies prior to multi-year El Niño events in observations. (**a–e**) The JFM(0) SLP anomalies for the five observed multi-year El Niño events, respectively. (**f**) Regressions of SLP anomalies onto the normalized JFM NPO index. (**g**) The normalized JFM(0) NPO index for the five observed multi-year El Niño events. The horizontal dashed line represents one positive standard deviation. (**h**) The spatial correlation coefficients of the five JFM(0) SLP patterns in (**a–e)** with the typical NPO pattern in (**f**) over the North Pacific region (15°–70°N and 150°E–120°W). The horizontal dashed line shows the 95% confidence level. The confidence level at which the spatial correlations are significant is calculated using an effective number of spatial degrees of freedom. The effective numbers of spatial degrees of freedom for the five JFM(0) SLP patterns are slightly different, and we have taken the minimum value.

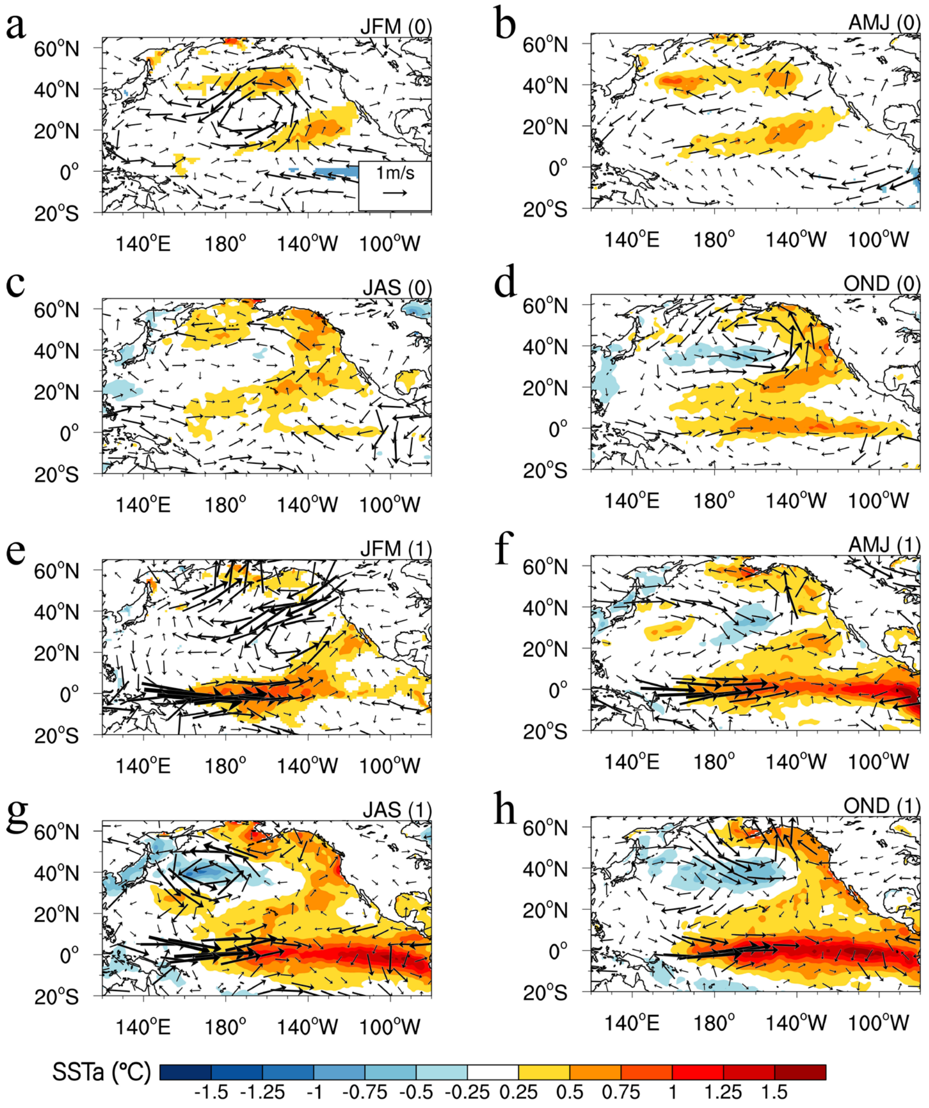

Supplementary Figure 4. Evolutions of SST and 850 hPa winds anomalies composited for multi-year El Niño events in observations. (**a–i**) Composite anomalies of 3-month-averaged SST (shaded) and 850 hPa winds (vector) for JFM(0) concurrent with the NPO index (**a**), and several lead times [AMJ(0) (**b**), JAS(0) (**c**), OND(0) (**d**), JFM(1) (**e**), AMJ(1) (**f**), JAS(1) (**g**), and OND(1) (**i**)]. Only SST and 850 hPa wind anomalies significant at the 90% confidence level are shown.

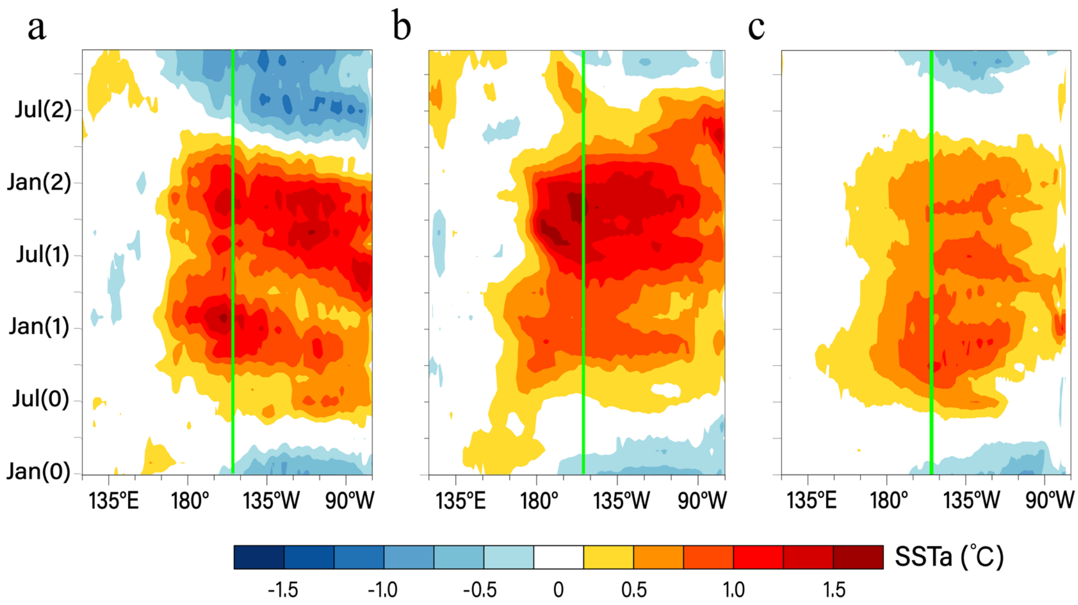

Supplementary Figure 5. Evolutions of SST anomalies composited for multi-year El Niño events in observations and models. (**a,b**) Longitude-time plot of equatorial (5°S–5°N) SST anomalies from year (0) to year (2) composited for the NPO-preceded multi-year El Niño events in observations and CMIP5/6 models (MME), respectively. (**c**) Ensemble-mean difference in equatorial (5°S–5°N) SST anomalies from year (0) to year (2) between the positive NPO forcing and CTRL experiments. In (**a,b,c**), the reference longitude of 155°W is indicated by the green vertical line, and only SST anomalies significant at the 95% confidence level are shown.

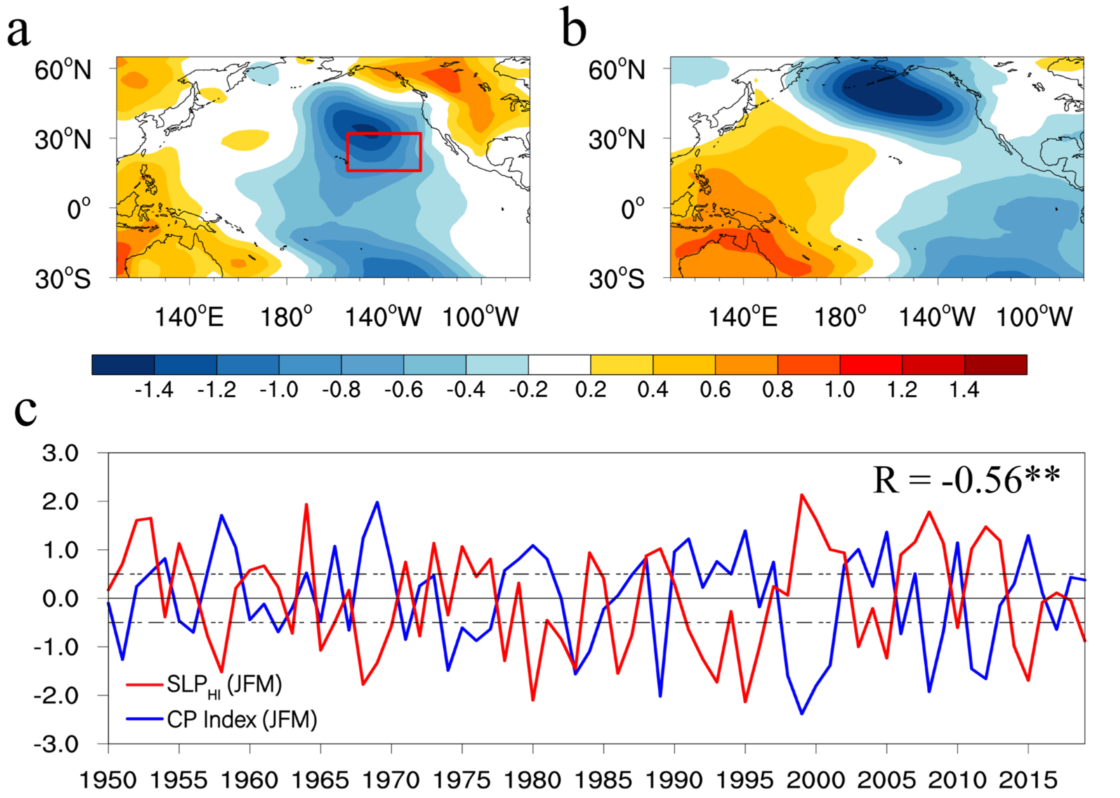

Supplementary Figure 6. The atmospheric teleconnections of the CP and EP ENSO. (**a**) Regressions of SLP anomalies (hPa) onto JFM values of the standardized CP ENSO index. (**b**) As in (**a**) but for regressions of SLP anomalies onto the standardized EP ENSO index. (**c**) Time series of the SLP index over the Hawaiian region (SLP_{HI}; 155°–125°W, 16°–32°N) (red line) and the CP ENSO index (black line). Indices are normalized by their standard deviations and are correlated R=-0.56 (significant at the 99.9% confidence level). In (**a,b**), only SLP anomalies significant at the 95% confidence level are shown.

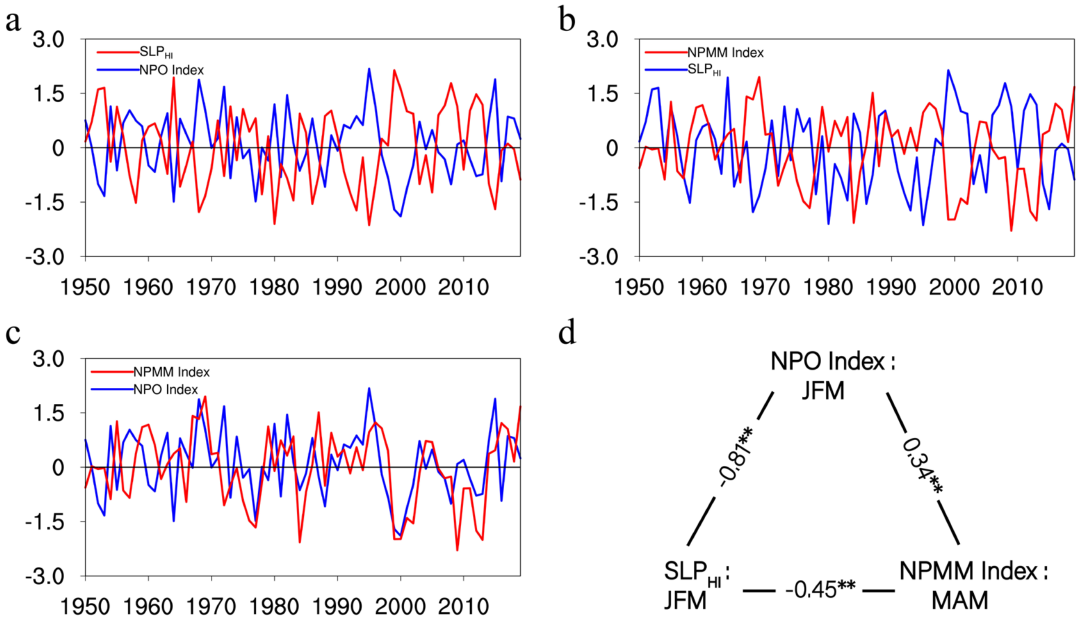

Supplementary Figure 7. Relationships among the NPO, SLP_{HI}, and NPMM indices. (a) Time series of the JFM NPO index and the JFM SLP index over the Hawaiian region (SLP_{HI}; 155°–125°W, 16°–32°N). (**b**) Time series of the JFM SLP_{HI} index and the following MAM NPMM index. (**c**) Time series of the JFM NPO index and the following MAM NPMM index. (**d**) Correlations among the NPO, SLP_{HI}, and NPMM indices, and ** indicates significant at the 99% confidence level.

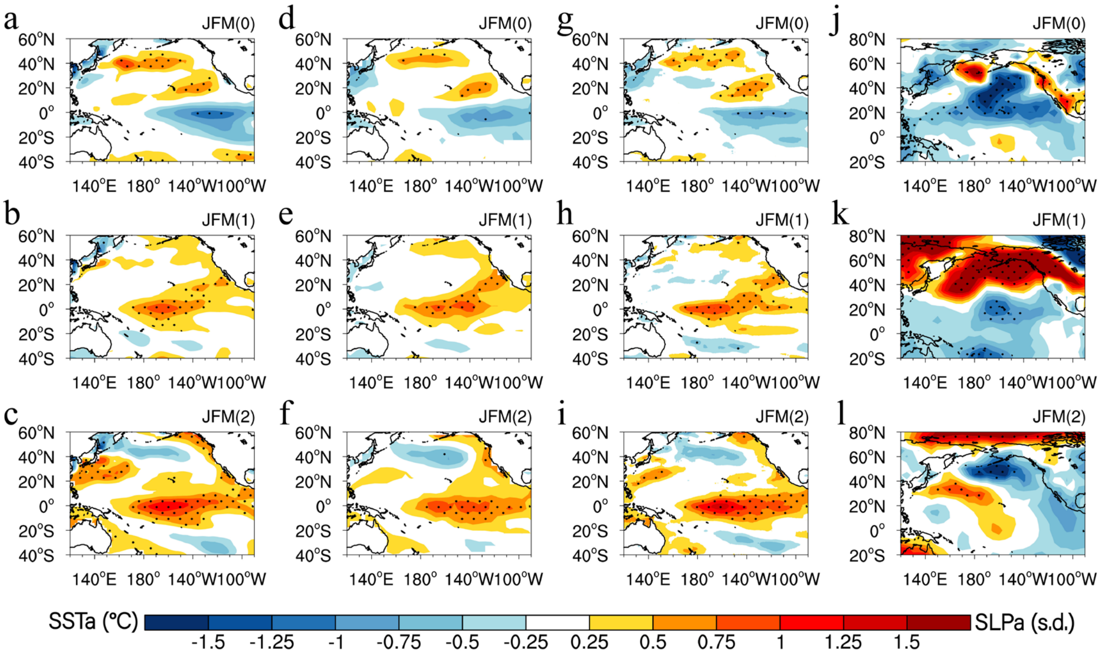

Supplementary Figure 8. Evolutions of SST and SLP anomalies composited for multi-year El Niño events from JFM(0) to JFM(2) derived from different SST and SLP datasets. (**a,b,c**) Evolutions of SST anomalies derived from the ERSST dataset. (**d,e,f**) Evolutions of SST anomalies derived from the Kaplan SST dataset. (**g,h,i**) Evolutions of SST anomalies derived from the COBE SST dataset. (**j,k,l**) Evolutions of SLP anomalies derived from the HadSLP dataset. In (**a–l**), dots indicate SST and SLP anomalies significant at the 95% confidence level.

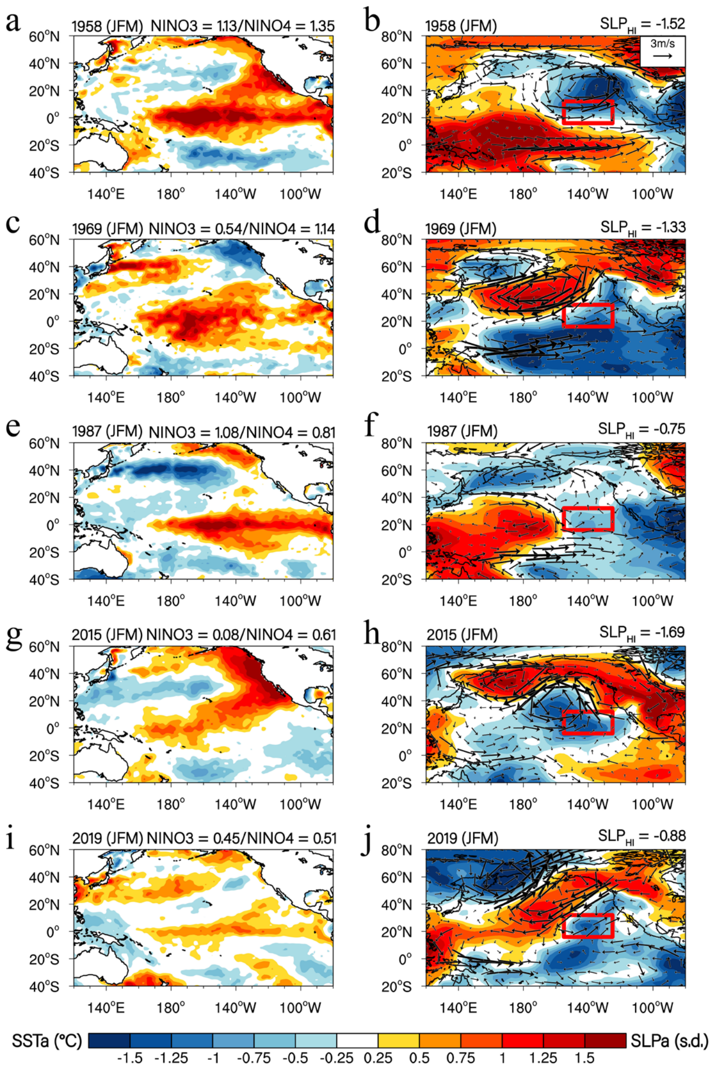

Supplementary Figure 9. The JFM(1) anomalies of SST, SLP and 850 hPa winds for individual multi-year El Niño events. (**a**) JFM 1958 SST (shaded) anomalies. (**b**) JFM 1958 SLP (shaded) and 850 hPa winds (vector) anomalies. (**c**) JFM 1969 SST (shaded) anomalies. (**d**) JFM 1969 SLP (shaded) and 850 hPa winds (vector) anomalies. (**e**) JFM 1987 SST (shaded) anomalies. (**f**) JFM 1987 SLP (shaded) and 850 hPa winds (vector) anomalies. (**g**) JFM 2015 SST (shaded) anomalies. (**h**) JFM 2015 SLP (shaded) and 850 hPa winds (vector) anomalies. (**i**) JFM 2019 SST (shaded) anomalies. (**j**) JFM 2019 SLP (shaded) and 850 hPa winds (vector) anomalies. In (**a,c,e,g,i**), the Niño3 and Niño4 indices are provided in the top right. For these 5 multi-year El Niño events, the Niño4 index is greater than or is comparable to the Niño3 index during JFM(1), indicating a typical CP El Niño pattern or a mixed pattern of CP and EP El Niño. In (**b,d,f,h,j**), the SLP index over the Hawaiian region (SLP_{HI}; 155°–125°W, 16°–32°N) is provided in the top right.

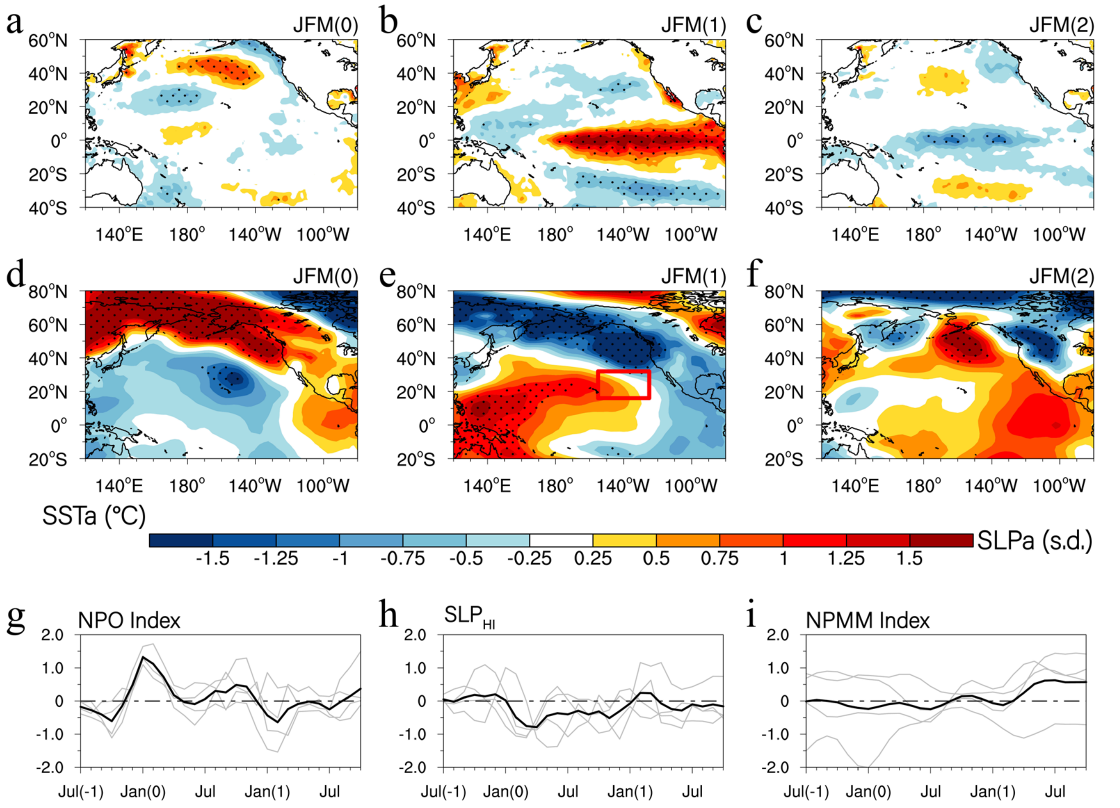

Supplementary Figure 10. Evolutions of SST and SLP anomalies composited for the NPO-preceded single-year El Niño events in observations. (**a,b,c**) JMF(0), JFM(1), and JFM(2) SST anomalies, respectively. (**d,e,f**) JMF(0), JFM(1), and JFM(2) SLP anomalies, respectively. (**g,h,i**) Temporal evolutions of the NPO index, the SLP index over the Hawaiian region (SLP_{HI}), and the NPMM index, respectively. The red box in (**e**) denotes region used to compute the SLP index over the Hawaiian region (SLP_{HI}; 155°–125°W, 16°–32°N). In (**a–f**), dots indicate SST and SLP anomalies significant at the 95% confidence level. In (**g,h,i**), the gray curves indicates individual evolutions of various indices.

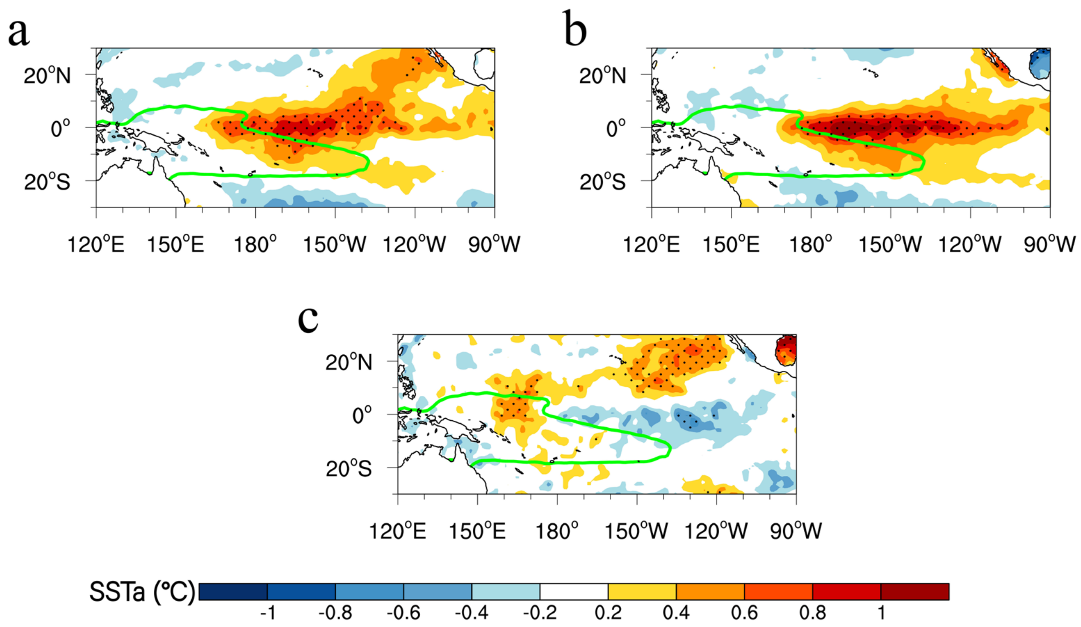

Supplementary Figure 11. Composite structures of JFM(1) SST anomalies for the NPO-preceded and non-NPO-preceded CP El Niño events. (**a**) Composite structures of JFM(1) SST anomalies for the NPO-preceded CP El Niño events. (**b**) As in (**a**), but for composite structures of JFM(1) SST anomalies for CP El Niño events alone without the preceding NPO events (non-NPO-preceded CP El Niño events). (**c**) Composite differences of JFM(1) SST anomalies between the NPO-preceded and non-NPO-preceded CP El Niño events. In (**a–c**), dots indicate SST anomalies significant at the 95% confidence level, and the green contours denote climatological 28°C isotherm line which depicts an area for the western Pacific warm pool during JFM.

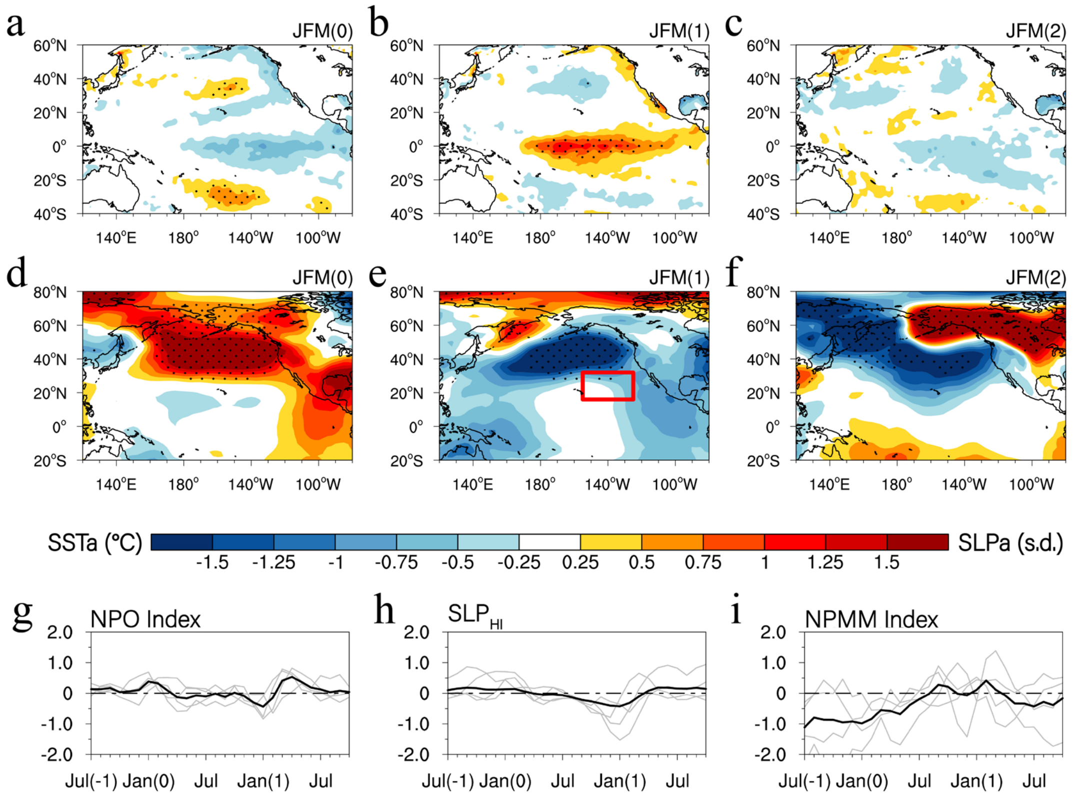

Supplementary Figure 12. Evolutions of SST and SLP anomalies composited for the observed CP El Niño events without the preceding NPO in observations. (**a,b,c**) JMF(0), JFM(1), and JFM(2) SST anomalies, respectively. (**d,e,f**) JMF(0), JFM(1), and JFM(2) SLP anomalies, respectively. (**g,h,i**) Temporal evolutions of the NPO index, the SLP index over the Hawaiian region (SLP_{HI}), and the NPMM index, respectively. The red box in (**e**) denotes region used to compute the SLP index over the Hawaiian region (SLP_{HI}; 155°–125°W, 16°–32°N). In (**a–f**), dots indicate SST and SLP anomalies significant at the 95% confidence level. In (**g,h,i**), the gray curves indicates individual evolutions of various indices.

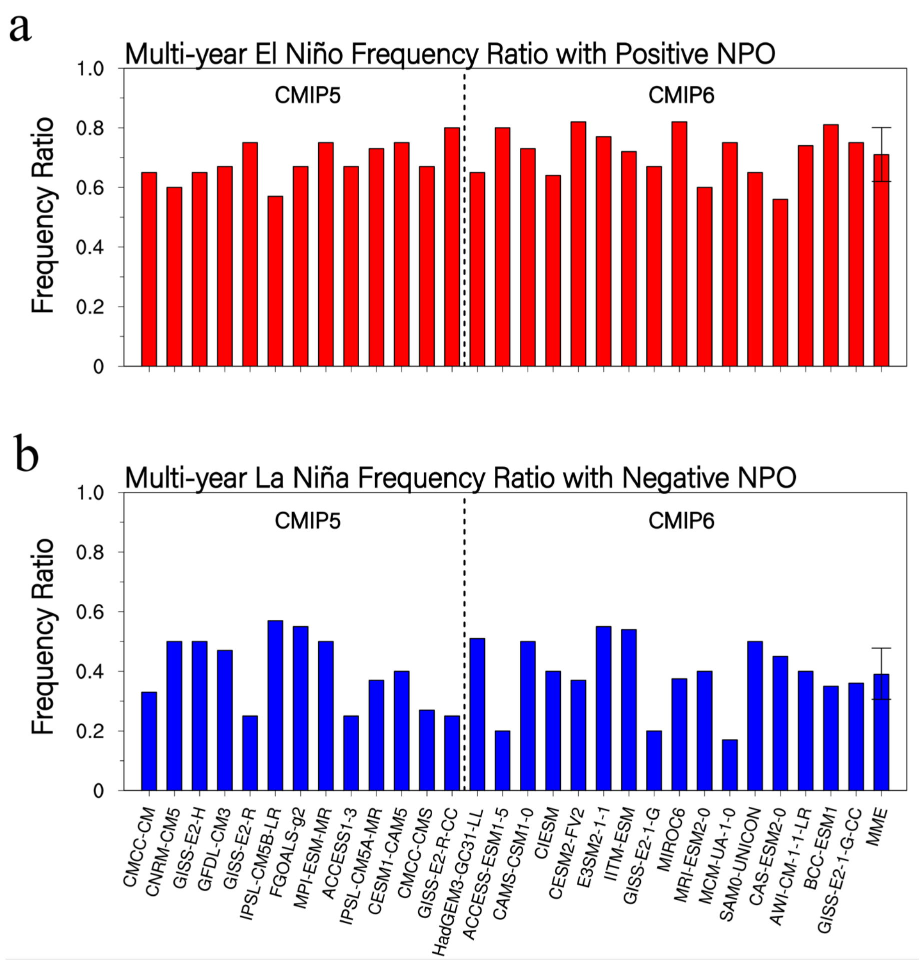

Supplementary Figure 13. Effects of the NPO on the frequency ratio of multi-year El Niño/La Niña events in the CMIP5/6 models. (a) The ratio of multi-year El Niño events preceded by positive NPO event during JFM(0). (**b**) As in (**a**) but for multi-year La Niña cases. Error bars in the multi-model mean correspond to the 95% confidence interval.

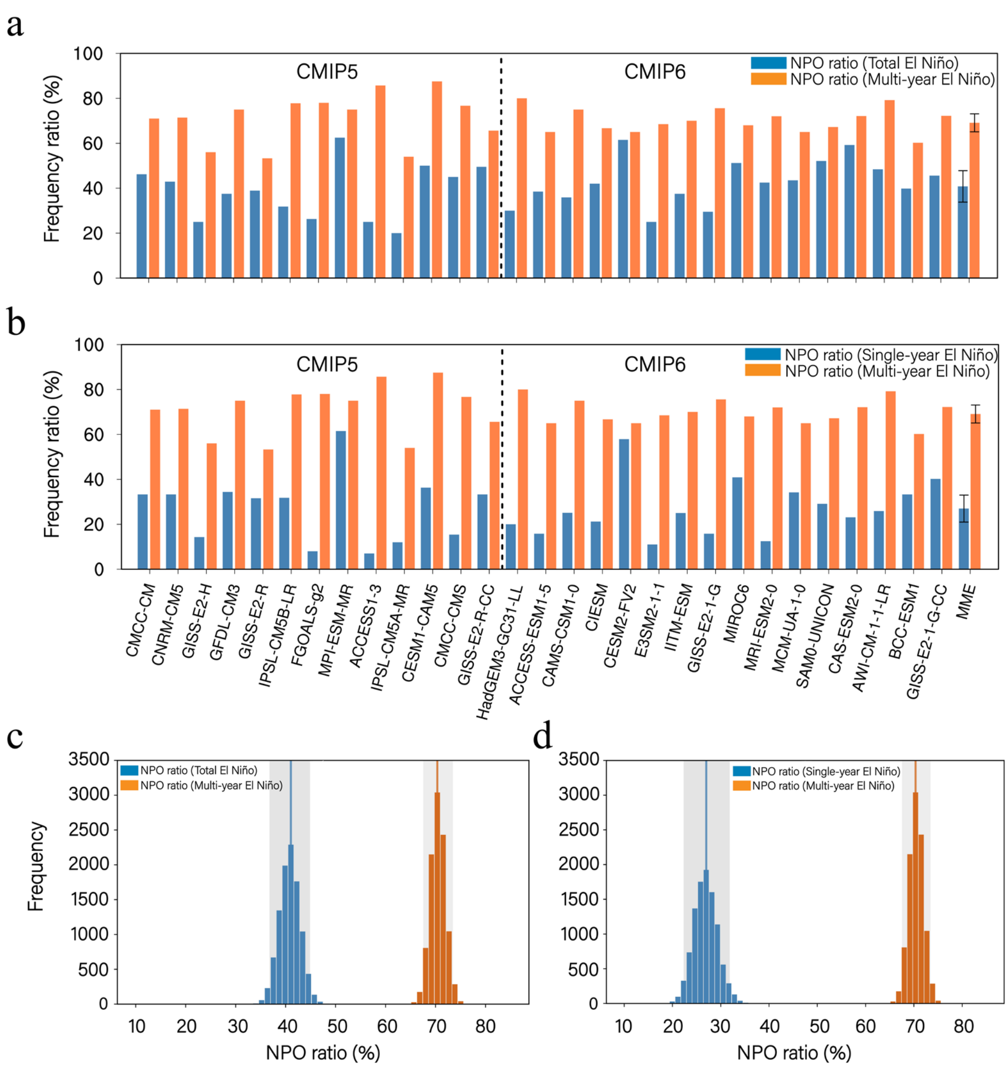

Supplementary Figure 14. The frequency ratio of the NPO-preceded El Niño/single-year El Niño events in the CMIP5/6 models. (**a**) The ratios of the NPO-preceded multi-year El Niño (red) and El Niño (including both single-year and multi-year El Niño, blue) events. Error bars in the multi-model mean indicate the 95% confidence interval. (**b**) As in (**a**) but for the ratios of the NPO-preceded multi-year El Niño (red) and single-year El Niño (blue) events. (**c**) Histograms of 10,000 realizations of a bootstrap method for the ratios of the NPO-preceded multi-year El Niño (red) and El Niño (blue) events. The red and blue lines indicate the mean values of the 10,000 realizations for the ratios of the NPO-preceded multi-year El Niño and El Niño events, respectively. The gray shaded areas refer to the respective 1.0 SD of the 10,000 realizations. (**d**) As in (**c**) but for histograms of 10,000 realizations of a bootstrap method for the ratios of the NPO-preceded multi-year El Niño (red) and single-year El Niño (blue) events.

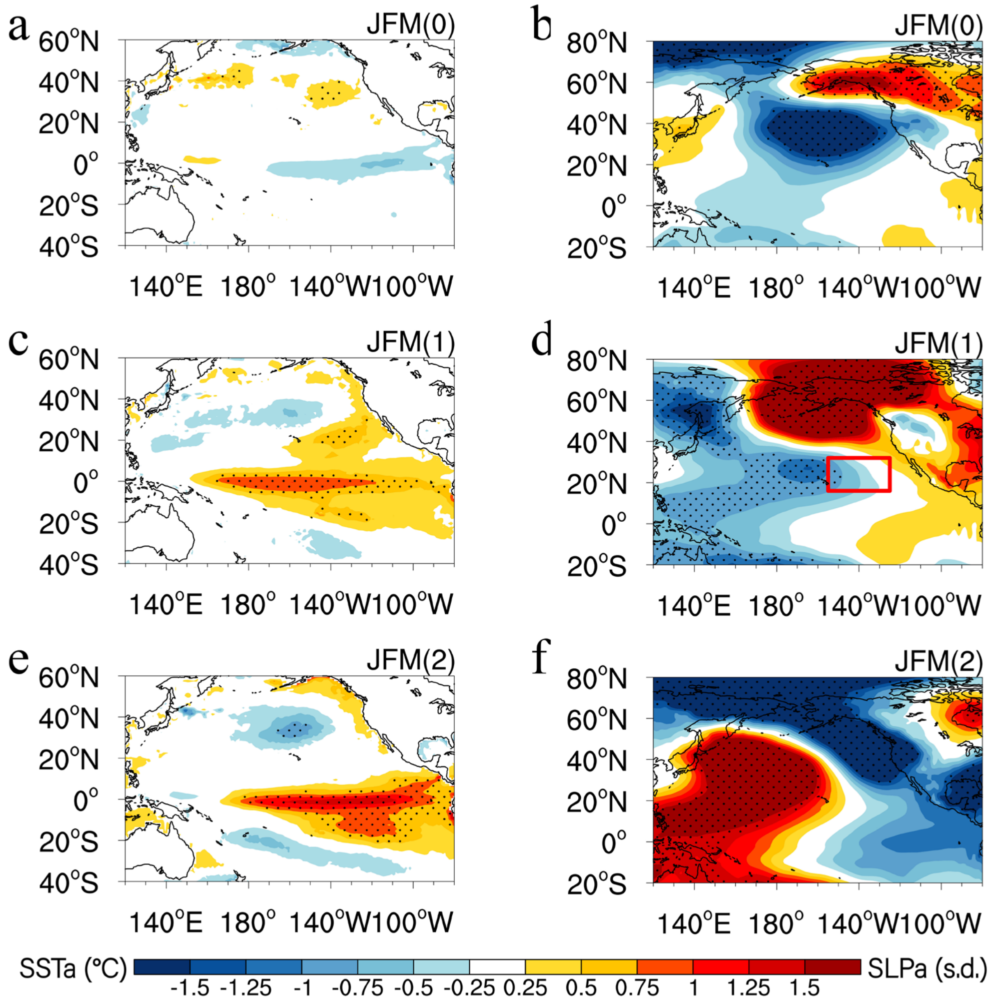

Supplementary Figure 15. Evolutions of SST and SLP anomalies composited for the NPO-preceded multi-year El Niño events in the CMIP5/6 models. (**a,b**) The JFM(0) SST and SLP anomalies, respectively. (**c,d**) The JFM(1) SST and SLP anomalies, respectively. (**e,f**) The JFM(2) SST and SLP anomalies, respectively. The red box in (**d**) denotes region used to compute the SLP index over the Hawaiian region (SLP_{HI}; 155°–125°W, 16°–32°N). In (**a–f**), dots indicate SST and SLP anomalies significant at the 95% confidence level.

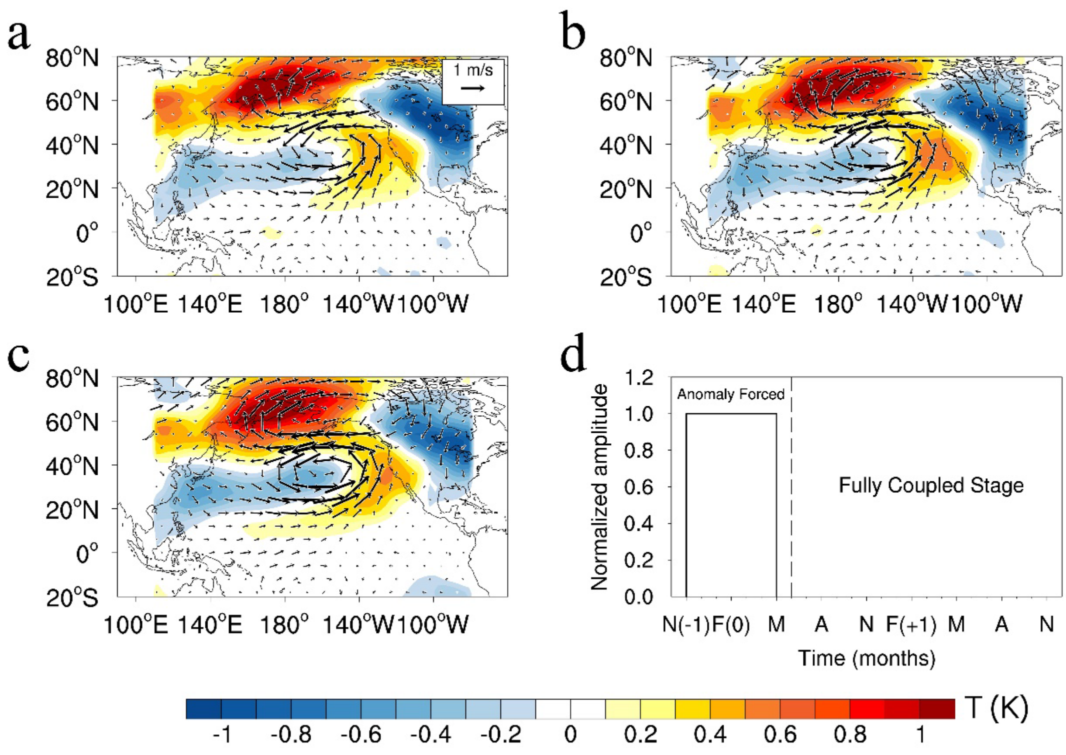

Supplementary Figure 16. The imposed NPO forcing in numerical experiments. (**a**) The spatial pattern of the NDJFMAM-averaged air temperature (K; shaded) and wind (m s^{-1}; vectors) forcings added to the climatological forcing and applied to each ensemble member in the NPO forcing experiment. The NDJFMAM-averaged air temperature and wind forcings are obtained by regressing air temperature and wind anomalies at 1000 hPa onto the concurrent NPO index. (**b**) As in (**a**) but for 925 hPa. (**c**) As in (**a**) but for 850 hPa. (**d**) Time evolution of the forcing applied during the assimilation stage (from December(−1) to May(0)) and the fully coupled stage of the NPO forcing experiment (from June(0) to February(1)).

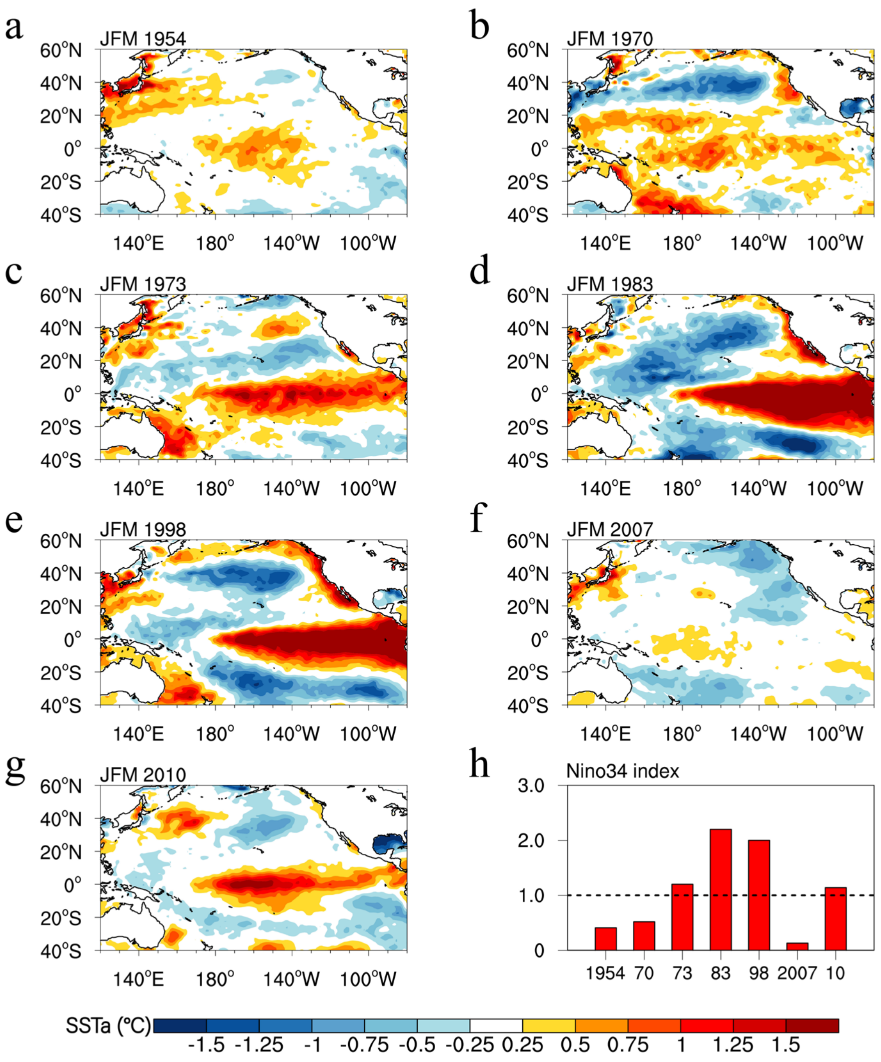

Supplementary Figure 17. The JFM(0) SST anomalies prior to multi-year La Niña events in observations. (**a–g**) The JFM(0) SST anomalies for the seven observed multi-year La Niña events. (**h**) The normalized JFM(0) Niño3.4 index for the seven observed multi-year La Niña events. The horizontal dashed line represents one positive standard deviation (SD). Four of the seven multi-year La Niña events (57%) are preceded by strong El Niño events (with the JFM(0) Niño3.4 index > 1.0 SD).

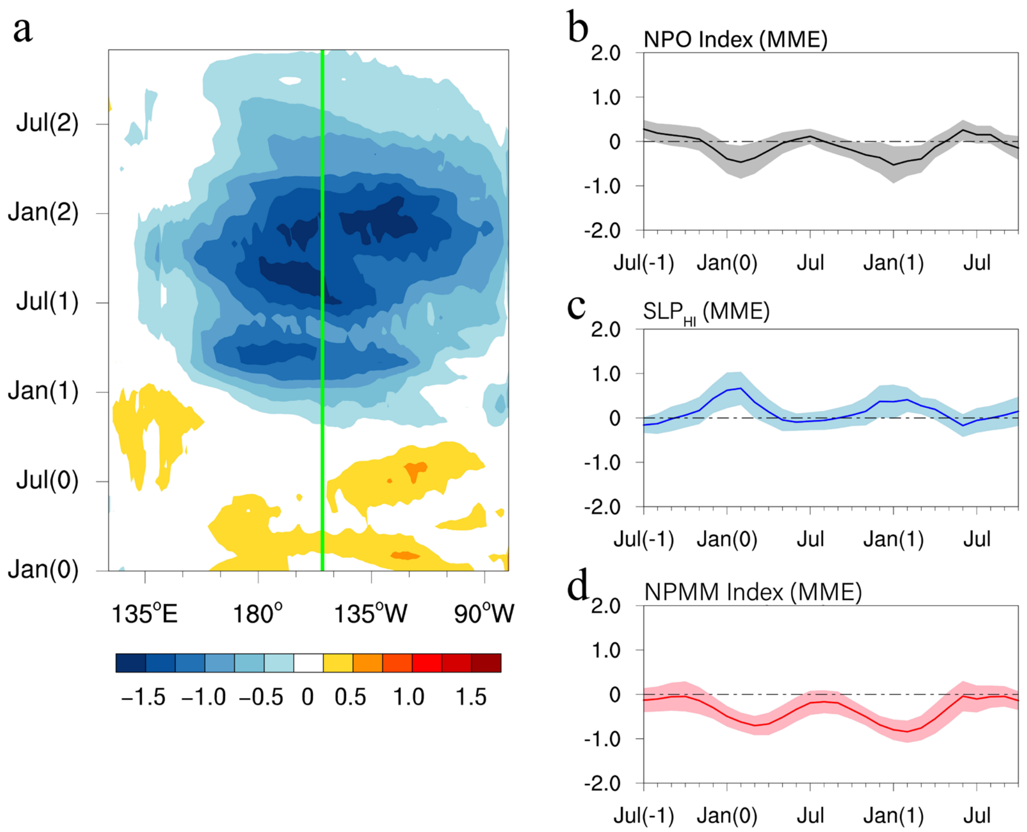

Supplementary Figure 18. Evolutions of multi-year La Niña events preceded by the negative NPO event alone in the CMIP5/6 models. (**a**) Longitude-time plot of equatorial SST anomalies from year (0) to year (2) composited for multi-year La Niña events. Only SST anomalies significant at the 95% confidence level are shown, and the reference longitude of 155°W is indicated by the green vertical line. (**b,c,d**) Temporal evolutions of the NPO index, the SLP index over the Hawaiian region (SLP_{HI}), and the NPMM index from July(−1) to October(1). In (**b,c,d**), the colored shading indicates interquartile ranges between the 25th and 75th percentiles.
